# Association between urinary albumin-to-creatinine ratio within normal range and kidney stones in U.S. adults: a cross-sectional observational study

**DOI:** 10.3389/fendo.2025.1526694

**Published:** 2025-03-14

**Authors:** Yuan-Zhuo Du, Chi-Teng Zhang, De-Ming Zeng, Yong Li, Yi-Fu Liu

**Affiliations:** ^1^ Department of Urology, The First Affiliated Hospital, Jiangxi Medical College, Nanchang University, Nanchang, China; ^2^ Jiangxi Institute of Urology, Nanchang, China; ^3^ The Second Affiliated Hospital, Department of Urology, Hengyang Medical School, University of South China, Hengyang, China

**Keywords:** urinary albumin-to-creatinine ratio, kidney stones, national health and nutrition examination survey, cross-sectional study, U.S. adults

## Abstract

**Background:**

Kidney stones are a major public health concern, and their prevalence has increased significantly in recent decades. While urinary albumin-to-creatinine ratio (UACR) is a recognized marker for kidney disease, its relationship with kidney stones, especially within the normal UACR range, remains unclear. The purpose of this study was to investigate the association between UACR levels within the normal range and the risk of developing kidney stones.

**Methods:**

We analyzed data from the National Health and Nutrition Examination Survey (NHANES) conducted from 2009 to 2018, focusing on adults aged 20 years and older with available UACR data. Using weighted multivariable logistic regression and restricted cubic spline (RCS) models, we assessed the relationship between UACR levels and the prevalence of kidney stones, adjusting for relevant covariates. Subgroup analyses were also performed to evaluate the consistency of this association across demographic and health-related factors.

**Results:**

The study found that higher UACR levels within the normal range were significantly associated with an increased likelihood of developing kidney stones. Specifically, individuals in the highest quartile of UACR had a 36% higher odds of kidney stones compared to those in the lowest quartile (OR: 1.36, 95% CI: 1.04-1.77). A non-linear, dose-response relationship was observed between UACR levels and kidney stone risk (P < 0.001), with the association remaining consistent across various demographic subgroups.

**Conclusion:**

Elevated UACR levels, even within the normal range, are strongly associated with a higher risk of kidney stones. This finding highlights the potential of UACR as a valuable biomarker for assessing kidney stone risk in clinical practice.

## 1 Introduction

Kidney stones are a significant public health issue globally, particularly prevalent in industrialized nations (1, 2). Over the past few decades in America, there has been a significant increase in the prevalence of kidney stones, impacting millions and adversely affecting their overall well-being (2, 3). The prevalence of kidney stones in the United States is approximately 9-10% of adults, with a growing incidence across various demographic groups, including men and women of different age groups, ethnicities, and socioeconomic statuses (4). The recurrence rate of kidney stones is notably high, with studies showing that up to 50% of individuals who have had a kidney stone will experience another episode within 10 years (1, 5, 6). This recurrence rate is particularly concerning given the painful nature of kidney stone episodes and the associated risk of chronic kidney disease (CKD) over time. The formation of kidney stones is influenced by a wide range of factors, including diet, genetics, hydration levels, and underlying health conditions (6–8). Increased dietary salt and oxalate intake, insufficient fluid consumption, and sedentary behavior are key contributors to the rising incidence (9–11). As a result, the recurrent nature of kidney stones further emphasizes the need for early identification, effective management, and preventive measures (6, 12).

Elevated levels of albumin in urine, known as albuminuria, are considered a warning sign of early renal impairment (13), and the urinary albumin-to-creatinine ratio (UACR) serves as a tool for monitoring the initial stages of chronic kidney disease (CKD) (14–16). Although a UACR within normal limits is typically considered indicative of healthy kidney function, recent studies have begun to focus on the health implications of minor variations within this range (17, 18). Specifically, increases in UACR within the normal range may reflect subtle, undetected renal impairments, which might be associated with a heightened risk of developing kidney stones (19, 20). Our analysis focuses on these minor fluctuations within the normal UACR range, which may have clinical significance for the early prevention and holistic treatment of kidney stones.

Here, based on data from the National Health and Nutrition Examination Survey (NHANES) from 2009 to 2018, we reveal the benefits of monitoring UACR within normal limits for the prevention and control of kidney stones on a public health scale. It offers crucial insights for the development of focused prevention strategies and the enhancement of clinical practices.

## 2 Methods

### 2.1 Study population

The study utilized information from the NHANES, a continuous program designed to evaluate the health and nutritional well-being of Americans. Around 5,000 individuals from the U.S. are involved each year, contributing information on their demographics, socioeconomic status, eating patterns, and health status. The data is gathered through in-person interviews and thorough physical assessments that include physiological measurements and lab tests. The process of obtaining ethical approval and informed consent was approved by the Institutional Review Board of the National Center for Health Statistics.

**Figure 1:**
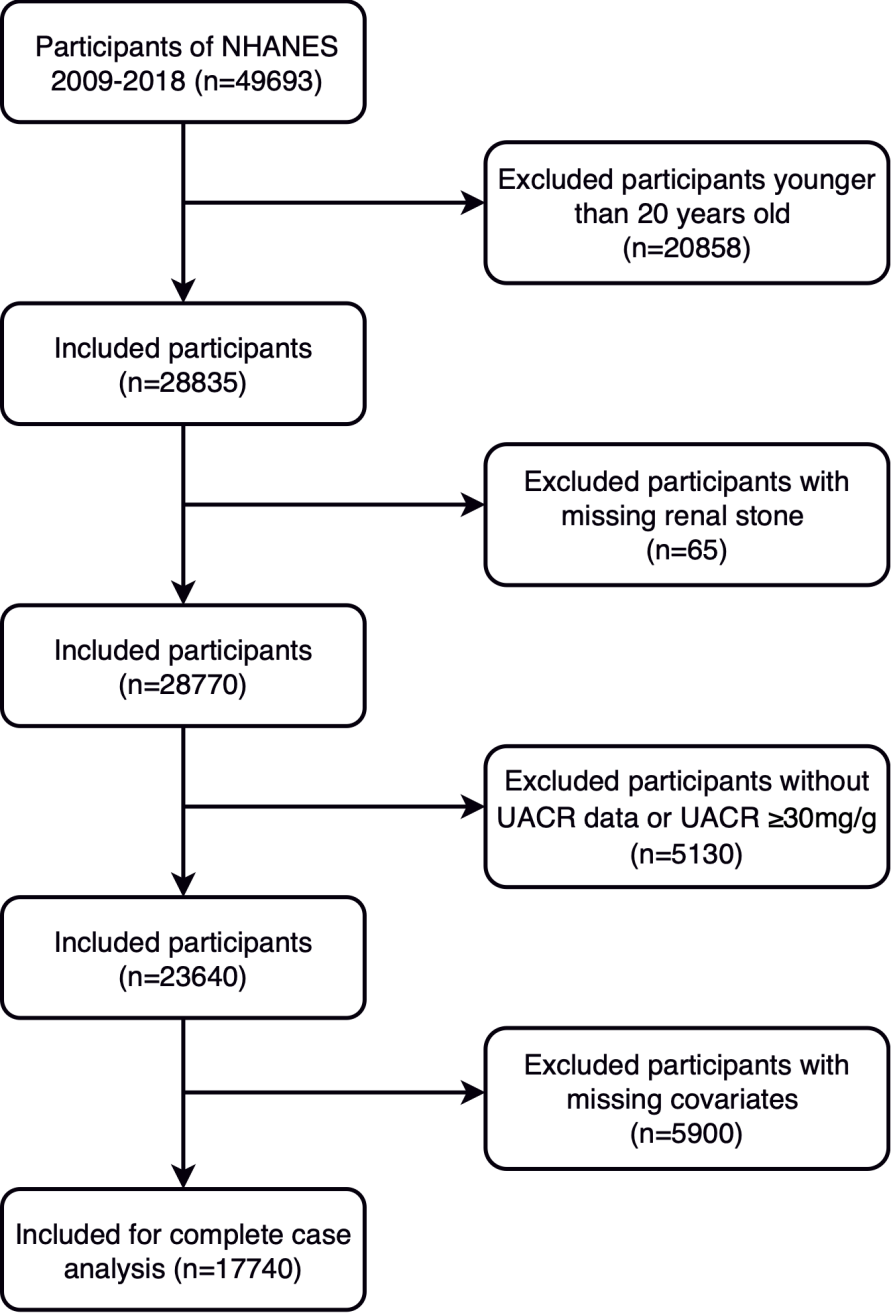
Flowchart for screening of participants.

In this observational study, data was collected from the NHANES database covering a period from 2009 to 2018. Initially, 49,693 potential participants were assessed based on predefined criteria for inclusion and exclusion. Participants were excluded if they were younger than 20 years old (20,858 individuals), lacked historical kidney stone data (65 individuals), had incomplete UACR data or UACR levels exceeding 30 mg/g (5,130 individuals), or were missing key covariate data (5,900 individuals). After applying these exclusions, a total of 17,740 individuals met the criteria and were included in the final dataset for analysis. For further specifics, please refer to **Figure 1**.

### 2.2 Measurement of UACR

Urine specimens were carefully prepared and stored at an ideal temperature of -30°C for further examination. The presence of urinary albumin was determined through a solid-phase fluorescence immunoassay technique, and the amount of urinary creatinine was measured using an enzymatic quantification method. The NHANES website provides a complete summary of the laboratory methodology (21). Following the NHANES guidelines, urinary albumin and creatinine levels underwent standardization and calibration utilizing the recognized gold standard approach. The UACR was presented in units of milligrams per gram (mg/g).

### 2.3 Diagnosis of kidney stones

The verification of the precision of participants’ self-reported history of kidney stones was achieved through inquiring, “Have you experienced kidney stones in the past?” This approach to confirmation is backed by existing research (22, 23). Consequently, individuals who affirmed the question were classified as having experienced kidney stones in the past.

### 2.4 Definition of covariates

The research incorporated various factors linked to UACR levels and the risk of kidney stone formation, divided into demographic, lifestyle, and health-related categories. Demographically, the study considered age, gender, ethnicity, marital status, education, and economic hardship. Lifestyle factors included alcohol consumption patterns (classified as never having consumed more than 12 drinks in their lifetime, former drinkers who had more than 12 drinks annually but not in the past year, and current drinkers who had more than 12 drinks in their lifetime and at least once in the past year) (24), smoking habits (determined by a history of smoking over 100 cigarettes), sedentary behavior (daily sitting time, excluding sleep, with categories for less than 5 hours and 5 hours or more), and physical exercise (assessed by the time spent on moderate to vigorous activities for at least 10 consecutive minutes daily, with inactivity defined as less than 10 minutes per day) (25). Sedentary behavior was defined based on a participant’s reported sitting time during activities such as sitting at school, at home, commuting, reading, watching television, or using a computer, excluding time spent sleeping. Health metrics encompassed the Body Mass Index (BMI), serum uric acid, the estimated glomerular filtration rate (eGFR), as well as conditions such as diabetes, hypertension, hyperlipidemia, and cardiovascular disease (CVD). These data were gathered utilizing standardized surveys and medical evaluations. The eGFR was calculated using the Chronic Kidney Disease Epidemiology Collaboration (CKD-EPI) equation (26):


$$\begin{array}{c} \text{Male: eGFR}=141\\ \times \min{\left(\frac{\text{Scr}}{0.9},1\right)}^{-0.411}\times \max{\left(\frac{\text{Scr}}{0.9},1\right)}^{-1.209}\times 0.993^{\text{Age}}\\ \times 1.159\left(\text{if black}\right) \end{array}$$

$$\begin{array}{c} \text{Female: eGFR}=141\\ \times \min{\left(\frac{\text{Scr}}{0.7},1\right)}^{-0.329}\times \max{\left(\frac{\text{Scr}}{0.7},1\right)}^{-1.209}\times 0.993^{\text{Age}}\\ \times 1.018\left(\text{if black}\right) \end{array}$$

Where SCr represents serum creatinine (mg/dL), and the values are adjusted based on the patient’s age, gender, and race (if Black).

### 2.5 Statistical analysis

Adhering to the NHANES protocol for sampling and weighting helped ensure that the sample was representative, with appropriate modifications to the weights. Specifically, we used the “Full sample 2year MEC exam weight” provided in the NHANES dataset. The statistical methods included analyzing continuous data (mean values and standard deviations) and categorical data (counts and proportions), using weighted linear regression for the former and chi-square tests for the latter.

We utilized a multivariable logistic regression analysis to explore the potential association between UACR and the risk of kidney stones. UACR was evaluated both as a continuous measure and in a categorized manner, divided into quartiles with the lowest quartile serving as the control group (18). For each regression model, we computed the odds ratios (ORs) along with their corresponding 95% confidence intervals (CIs). The crude model was not adjusted for any covariates, whereas Model 1 included adjustments for age, gender, ethnicity, educational attainment, marital status, and socioeconomic status. Model 2 further incorporated adjustments for smoking habits, alcohol intake, BMI, serum uric acid, eGFR, sedentary lifestyle, exercise levels, hypertension, diabetes, hyperlipidemia, and CVD. To delve into the potential dose-response relationship between UACR and the likelihood of kidney stones, we employed restricted cubic spline (RCS) regression analysis. Additionally, subgroup analyses were conducted to verify the consistency of our results across different demographic and health-related variables. To assess the predictive ability of UACR for kidney stone formation, we conducted a receiver operating characteristic (ROC) curve analysis. The area under the curve (AUC) was calculated to evaluate the discriminative ability of UACR for distinguishing between individuals with and without kidney stones. All statistical computations were performed using R software, version 4.3.2, with statistical significance defined as P<0.05.

## 3 Results

### 3.1 Baseline characteristics of participants

In this analysis, data from 17,740 participants spanning five NHANES cycles from 2009 to 2018 were examined. The study categorized participants into four groups based on their UACR. **Table 1** outlines the demographic characteristics of the cohort, with a mean age of 46.44 years (± 0.30), 50.75% female participants, and a weighted prevalence of kidney stones of 9.64%. The findings suggest that individuals with elevated UACR values, even within what is considered normal, are more likely to be older, predominantly female, of Mexican American ethnicity, unmarried (including divorced, separated, or widowed), have lower educational levels, lower income ratios, lower BMI, lower serum uric acid level, be smokers, non-drinkers, lead sedentary lifestyles, and have a history of hypertension, diabetes, hyperlipidemia, and CVD. Additionally, there is a significant correlation between higher UACR levels and a higher likelihood of developing kidney stones.

**Table 1:** Baseline characteristics of the study population by quartiles of UACR.

Variable	UACR (mg/g) quartiles					
	Overall	Quartile 1	Quartile 2	Quartile 3	Quartile 4	P-value
Age, y, mean (SE)	46.44 (0.30)	42.06 (0.41)	45.40 (0.44)	47.77 (0.43)	51.50 (0.40)	<0.0001
Age strata, y, n (%)						<0.0001
20–39	6484 (37.99)	2123 (47.57)	1737 (39.05)	1467 (34.70)	1157 (28.74)	
40–59	6079 (38.08)	1571 (38.59)	1592 (39.76)	1501 (37.74)	1415 (35.86)	
≥60	5177 (23.93)	769 (13.84)	1102 (21.19)	1447 (27.56)	1859 (35.40)	
Sex, n (%)						<0.0001
Female	9024 (50.75)	1445 (31.56)	2246 (51.18)	2624 (59.71)	2709 (63.73)	
Male	8716 (49.25)	3018 (68.44)	2185 (48.82)	1791 (40.29)	1722 (36.27)	
Race, n (%)						<0.001
Mexican American	2498 (8.06)	521 (7.18)	635 (8.05)	686 (8.45)	656 (8.72)	
Non-Hispanic White	7610 (68.95)	1879 (68.17)	1925 (69.57)	1907 (69.72)	1899 (68.32)	
Non-Hispanic Black	3522 (9.84)	1078 (11.81)	847 (9.11)	750 (8.37)	847 (9.90)	
Other Hispanic	1782 (5.55)	398 (5.34)	457 (5.71)	446 (5.39)	481 (5.78)	
Other Race	2328 (7.60)	587 (7.50)	567 (7.55)	626 (8.07)	548 (7.29)	
Marital status, n (%)						<0.0001
Divorced/Separated/Widowed	3599 (17.10)	604 (11.29)	811 (15.79)	987 (18.87)	1197 (23.73)	
Married/Living with a partner	10694 (64.21)	2772 (65.73)	2727 (65.76)	2646 (63.63)	2549 (61.18)	
Never married	3447 (18.69)	1087 (22.97)	893 (18.45)	782 (17.50)	685 (15.09)	
Education levels, n (%)						<0.0001
High school and below	7507 (34.88)	1728 (32.86)	1792 (33.08)	1921 (35.59)	2066 (38.65)	
Above high school	10233 (65.12)	2735 (67.14)	2639 (66.92)	2494 (64.41)	2365 (61.35)	
Poverty ratio, n (%)						<0.0001
<1.3	5459 (20.34)	1285 (18.88)	1323 (19.10)	1396 (21.06)	1455 (22.77)	
1.3-3.5	6576 (35.13)	1591 (33.11)	1586 (33.86)	1655 (35.77)	1744 (38.37)	
>3.5	5705 (44.53)	1587 (48.01)	1522 (47.04)	1364 (43.17)	1232 (38.86)	
BMI, n (%)						<0.0001
<18.5	243 (1.34)	34 (0.61)	48 (0.96)	57 (1.43)	104 (2.59)	
18.5-24.99	4843 (27.69)	1157 (25.44)	1246 (28.31)	1271 (28.63)	1169 (28.69)	
25-29.99	5868 (33.25)	1635 (37.53)	1520 (35.23)	1353 (31.32)	1360 (27.88)	
≥30	6786 (37.71)	1637 (36.43)	1617 (35.50)	1734 (38.62)	1798 (40.84)	
Smoke, n (%)						0.01
No	10105 (57.12)	2579 (58.74)	2577 (58.59)	2498 (56.14)	2451 (54.49)	
Yes	7635 (42.88)	1884 (41.26)	1854 (41.41)	1917 (43.86)	1980 (45.51)	
Alcohol user, n (%)						<0.0001
Never	2365 (9.89)	460 (8.13)	561 (9.81)	641 (10.44)	703 (11.53)	
Former	2372 (11.01)	512 (9.68)	574 (10.85)	605 (11.46)	681 (12.32)	
Now	13003 (79.10)	3491 (82.19)	3296 (79.34)	3169 (78.10)	3047 (76.15)	
Moderate recreational activity, n (%)						<0.0001
No	8561 (42.92)	1872 (38.09)	2041 (41.06)	2202 (43.55)	2446 (50.27)	
Yes	9179 (57.08)	2591 (61.91)	2390 (58.94)	2213 (56.45)	1985 (49.73)	
Sitting time, n (%)						0.61
<5	6856 (35.10)	1717 (35.05)	1738 (36.03)	1726 (35.11)	1675 (34.09)	
≥5	10884 (64.90)	2746 (64.95)	2693 (63.97)	2689 (64.89)	2756 (65.91)	
Hypertension, n (%)						<0.0001
No	11947 (70.52)	3415 (77.73)	3169 (73.97)	2874 (68.59)	2489 (59.84)	
Yes	5793 (29.48)	1048 (22.27)	1262 (26.03)	1541 (31.41)	1942 (40.16)	
Diabetes, n (%)						<0.0001
No	15544 (90.21)	4147 (94.52)	4000 (91.90)	3827 (89.46)	3570 (83.81)	
Borderline	430 (2.08)	86 (1.77)	98 (1.88)	113 (2.36)	133 (2.38)	
Yes	1766 (7.72)	230 (3.71)	333 (6.22)	475 (8.18)	728 (13.81)	
Hyperlipidemia, n (%)						<0.0001
No	5406 (31.12)	1633 (36.35)	1394 (32.16)	1276 (29.81)	1103 (25.00)	
Yes	12334 (68.88)	2830 (63.65)	3037 (67.84)	3139 (70.19)	3328 (75.00)	
CVD, n (%)						<0.0001
No	17355 (98.30)	4391 (98.96)	4362 (98.93)	4323 (98.40)	4279 (96.67)	
Yes	385 (1.70)	72 (1.04)	69 (1.07)	92 (1.60)	152 (3.33)	
eGFR (mL/min), mean (SE)	95.51 (0.39)	95.03 (0.49)	97.08 (0.56)	96.13 (0.56)	93.60 (0.51)	<0.0001
Uric acid (µmol/L), mean (SE)	320.59 (0.91)	339.95 (1.61)	315.35 (1.64)	311.82 (1.74)	312.79 (1.70)	<0.0001
Kidney stone, n (%)						<0.001
No	16138 (90.36)	4150 (92.13)	4073 (91.27)	3993 (89.26)	3922 (88.35)	
Yes	1602 (9.64)	313 (7.87)	358 (8.73)	422 (10.74)	509 (11.65)	

UACR, urinary albumin-to-creatinine ratio; eGFR, Estimated Glomerular Filtration Rate; BMI, Body mass index; CVD, Cardiovascular disease.

### 3.2 Relationship between UACR and kidney stones

**Figure 2:**
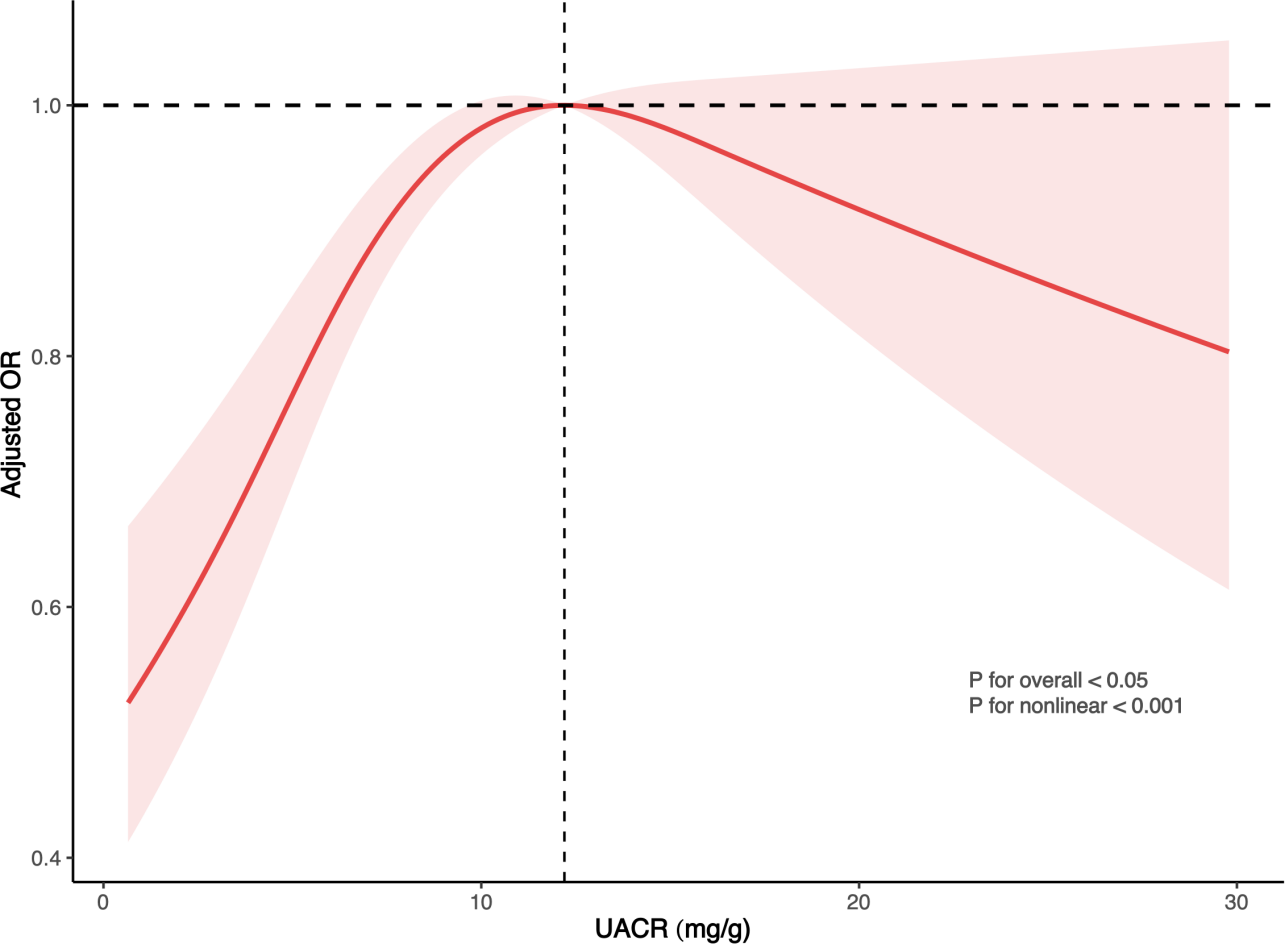
Illustration highlighting the relationship between UACR levels and the risk of developing kidney stones. The ORs, depicted by solid lines, have been adjusted to account for various factors including gender, age, ethnicity, educational attainment, marital status, economic hardship, BMI, uric acid, smoking habits, alcohol consumption, physical activity, sedentary behavior, eGFR, presence of hypertension, diabetes, hyperlipidemia, and CVD. Additionally, the 95% CIs, represented by shaded regions, have been considered to provide a comprehensive view of the data.

**Table 2** reveals a substantial positive link between UACR levels and the prevalence of kidney stones, assessed in both continuous and categorical manners. Initial unadjusted calculations showed that a 1 mg/g increment in UACR was associated with a 3% rise in the probability of kidney stone formation (95% CI: 1.01-1.04, P < 0.0001). This correlation persisted as significant in Model 2, which was comprehensively adjusted, yielding an odds ratio (OR) of 1.01 (95% CI: 1.00-1.03, P = 0.04). Within the fully adjusted quartile analysis, the risk escalated by 35% for the third quartile (95% CI: 1.08-1.70, P = 0.01) and by 36% for the fourth quartile (95% CI: 1.04-1.77, P = 0.02) when contrasted with the lowest quartile. Furthermore, employing a RCS regression analysis, a potential dose-response relationship was identified, characterized by a U-shaped distribution with an inflection point at 12.2 (**Figure 2**).

**Table 2:** Association of the quartiles of UACR with kidney stone.

Exposure	Crude model		Model 1		Model 2	
	OR (95% CI)	P value	OR (95% CI)	P value	OR (95% CI)	P value
UACR	1.03 (1.01,1.04)	<0.0001	1.02 (1.00,1.03)	0.01	1.01 (1.00,1.03)	0.04
UACR quartile						
Quartile1 [0.25,4.32]	1 (Ref.)		1 (Ref.)		1 (Ref.)	
Quartile2 (4.32,6.34]	1.12 (0.92,1.37)	0.26	1.10 (0.90,1.34)	0.36	1.12 (0.91,1.38)	0.28
Quartile3 (6.34,10.11]	1.41 (1.14,1.73)	0.002	1.35 (1.09,1.68)	0.01	1.35 (1.08,1.70)	0.01
Quartile4 (10.11,29.91]	1.54 (1.23,1.94)	<0.001	1.40 (1.10,1.79)	0.01	1.36 (1.04,1.77)	0.02
P for trend		<0.0001		<0.001		0.01

Crude model: unadjusted model.

Model 1: Adjusted for age, sex, race, education levels, marital status, poverty ratio.

Model 2: Additionally adjusted for BMI, smoking, alcohol user, recreational activity, sitting time, eGFR, uric acid, hypertension, diabetes, hyperlipidemia and CVD.

UACR, urinary albumin-to-creatinine ratio; OR, odds ratio; CI, confidence interval.

### 3.3 Subgroup analyses

**Figure 3:**
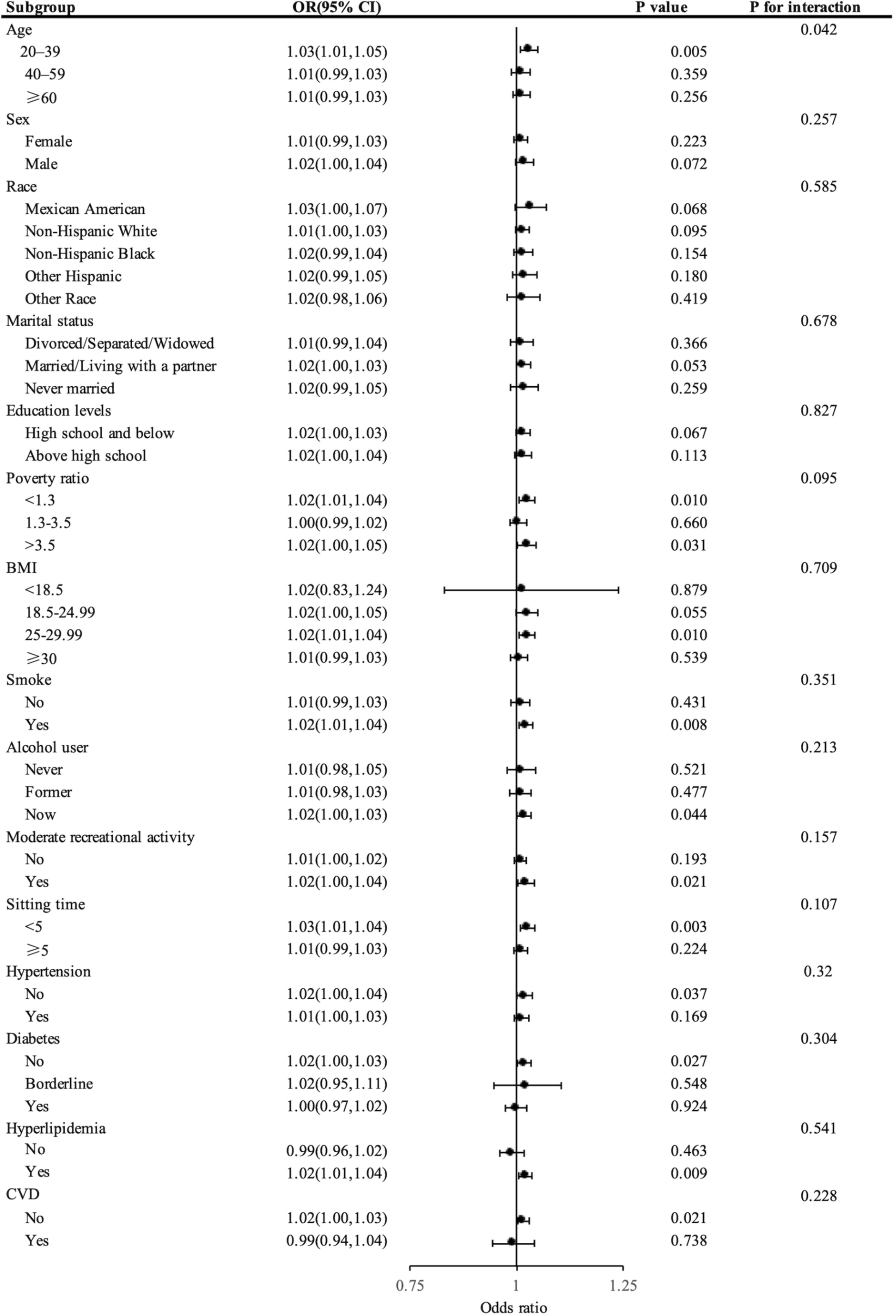
Forest plot showing stratified analysis of the correlation between UACR levels and the risk of developing kidney stones.

**Figure 3** presents the stratified analysis outcomes, highlighting a noticeable positive correlation between UACR and the prevalence of kidney stones across diverse population segments. Particularly, in individuals aged 20-40 years (OR = 1.03, 95% CI: 1.01-1.05), with lower (OR = 1.02, 95% CI: 1.01-1.04) or higher (OR = 1.02, 95% CI: 1.00-1.05) income poverty ratios, BMI ranging from 25-29.99 (OR = 1.02, 95% CI: 1.01-1.04), smokers (OR = 1.02, 95% CI: 1.01-1.04), current alcohol consumers (OR = 1.02, 95% CI: 1.00-1.03), those engaging in frequent physical activity (OR = 1.02, 95% CI: 1.00-1.04), those with shorter sitting durations (OR = 1.03, 95% CI: 1.01-1.04), and those without a history of hypertension (OR = 1.02, 95% CI: 1.00-1.04), those free from diabetes (OR = 1.02, 95% CI: 1.00-1.03), those without CVD (OR = 1.02, 95% CI: 1.00-1.03) and those diagnosed with hyperlipidemia (OR = 1.02, 95% CI: 1.01-1.04), the correlation was notably stronger, with all *P*-values below 0.05. Furthermore, significant interactions between UACR and kidney stone risk were noted in different age brackets (P *<* 0.05), whereas no such interactions were observed in other demographic groups (P *>* 0.05). Additionally, the estimates consistently revealed the same trend across all subgroups.

### 3.4 ROC curve analysis

To further assess the predictive ability of UACR for kidney stone formation, we conducted a ROC curve analysis. The AUC for the ROC curve was 0.5527, which suggests a moderate ability of UACR to discriminate between individuals with and without kidney stones (**Supplementary Figure 1**).

## 4 Discussion

The research draws on data from the NHANES to explore the potential link between normal levels of UACR and the likelihood of developing kidney stones. The findings suggest that even a minor increase in UACR within the normal range is associated with a higher risk of kidney stones, especially in individuals in the third and fourth quartiles when compared to those in the lowest quartile. The application of RCS regression models uncovered a non-linear, inverted U-shaped correlation between UACR and the risk of kidney stones, indicating a dose-response relationship. Additionally, this robust positive association was observed across various populations, underscoring the consistency and reliability of our findings.

The formation of kidney stones is a complex phenomenon involving multiple biochemical processes, including supersaturation of solutes in urine, nucleation, growth, aggregation, and eventual deposition on renal tissue (27–29). Minor urinary excretion of albumin may reflect subtle physiological changes in the kidneys that could indirectly promote stone formation by altering the biochemical environment of urine (30). For example, minor tubular damage could lead to protein leakage into the urine, potentially altering urine pH, which influence stone formation (31). Additionally, minor renal function changes may alter the concentration of inhibitory substances like citrate (32), which typically help prevent stone formation. Thus, even normal-range variations in UACR could significantly impact the risk of kidney stones.

Further studies indicate that patients with higher UACR have elevated inflammation scores (33), which suggests that increased UACR is not only a marker of CKD but also a possibly underrecognized risk factor for kidney stone formation (34, 35). Inflammatory processes may increase the permeability of renal tubules and surrounding tissues, facilitating more albumin to enter the urine (36). This increased protein excretion could activate proximal tubular epithelial cells, promoting the expression of, chemokines, and cytokines, further influencing the stone formation process (37). Additionally, the kidneys might indirectly affect stone formation probabilities by regulating urine pH and solute concentrations (38). The increase in UACR may reflect a subtle imbalance in renal function, particularly in handling calcium and phosphate, which could lead to increased urinary concentrations of these minerals and ultimately promote stone formation (28, 32). Moreover, renal function impairment might affect the activation of vitamin D (39), influencing calcium absorption and bone health, which may indirectly increase the risk of kidney stones (40).

Therefore, monitoring UACR could be valuable not only for assessing the risk of CKD but also for its potential utility in predicting kidney stone risk. This understanding underscores the importance of adopting a more comprehensive assessment approach in clinical practice to better prevent and manage kidney diseases such as kidney stones. Our study suggests that even individuals with UACR within the normal range but at higher normal values should undergo regular monitoring and possibly further evaluation and intervention, such as dietary adjustments, increased water intake, and appropriate medication, to reduce the formation of kidney stones. This emphasizes the relevance of UACR as a biomarker in public health and clinical practice.

A significant advantage of this research is the utilization of NHANES, a nationwide, representative dataset that offers crucial insights into the link between UACR levels and the likelihood of kidney stone formation. This large-scale population base offers sufficient statistical power to explore the potential links between UACR and kidney stone risk. Moreover, our use of RCS regression models allows us to explore the non-linear relationships between UACR and kidney stone risk, an aspect not extensively covered in previous studies.

However, the study is constrained by its cross-sectional design, preventing causal inferences. Cross-sectional studies can only capture data at one point in time, making it uncertain whether heightened UACR levels are a cause or a consequence of elevated kidney stone risk. Additionally, the study relies on self-reported history of kidney stones, which may be subject to recall bias. This could potentially lead to misclassification or underreporting of the true prevalence of kidney stones. Furthermore, the study does not capture incident cases or clinically confirmed diagnoses, which may result in underestimation of the true prevalence. The study also relies on a single urine measurement per participant, and variations in UACR levels within the normal range could affect the reliability and generalizability of the results. Although we adjusted for various covariates, there may be additional unmeasured factors such as dietary intake, genetic predispositions, or specific medications that could influence the risk of kidney stones. These unaccounted factors could introduce potential confounding or bias. Future research should adopt a prospective cohort design and perform multiple UACR measurements to more accurately determine the causal relationship between UACR and kidney stones. Additionally, considering the multifactorial nature of stone formation involving various environmental, dietary, and genetic factors, future studies should also consider these potential confounders to fully understand the association between UACR and the risk of kidney stones.

## 5 Conclusions

This study reveals a significant connection between elevated UACR levels within the normal range and an augmented risk of kidney stones, highlighting the potential value of monitoring UACR levels in the prevention and control of kidney stones. These findings provide new perspectives and evidence for future research and clinical practice.

## Data Availability

Publicly available datasets were analyzed in this study. This data can be found here: https://www.cdc.gov/nchs/nhanes/index.htm.
