# Supplementary material for: Association between urinary albumin-to-creatinine ratio within normal range and kidney stones in U.S. adults: a cross-sectional observational study
Source: Front Endocrinol (Lausanne). 2025 Mar 14;16:1526694. doi: 10.3389/fendo.2025.1526694 (PMC11949821; doi:10.3389/fendo.2025.1526694)
Supplement: Supplementary file 1 [file DataSheet1.docx]

**Supplementary Material**

**Association Between Urinary Albumin-to-Creatinine Ratio Within Normal Range and Kidney Stones in U.S. Adults: A Cross-Sectional Observational Study**

Yuan-Zhuo Du^1,2#^, Chi-Teng Zhang^3#^, De-Ming Zeng^3^, Yong Li^3*^, Yi-Fu Liu^3*^

^1^ Department of Urology, The First Affiliated Hospital, Jiangxi Medical College, Nanchang University, Nanchang 330000, Jiangxi Province, China

^2^ Jiangxi Institute of Urology, Nanchang 330000, Jiangxi Province, China.

^3^ The Second Affiliated Hospital, Department of Urology, Hengyang Medical School, University of South China, Hengyang, Hunan, China.

^#^ Yuan-Zhuo Du and Chi-Teng Zhang contributed equally to this work

* Correspondence: Yong Li^3*^, Yi-Fu Liu^3*^

^3^ The Second Affiliated Hospital, Department of Urology, Hengyang Medical School, University of South China, Hengyang, Hunan, China.

Yong Li^3*^ Email: liyong0724@126.com

Yi-Fu Liu^3*^ Email: lyf137556057762022@163.com

**Supplementary Figure 1:** ROC curves of the UACR for predicting kidney stones.

**
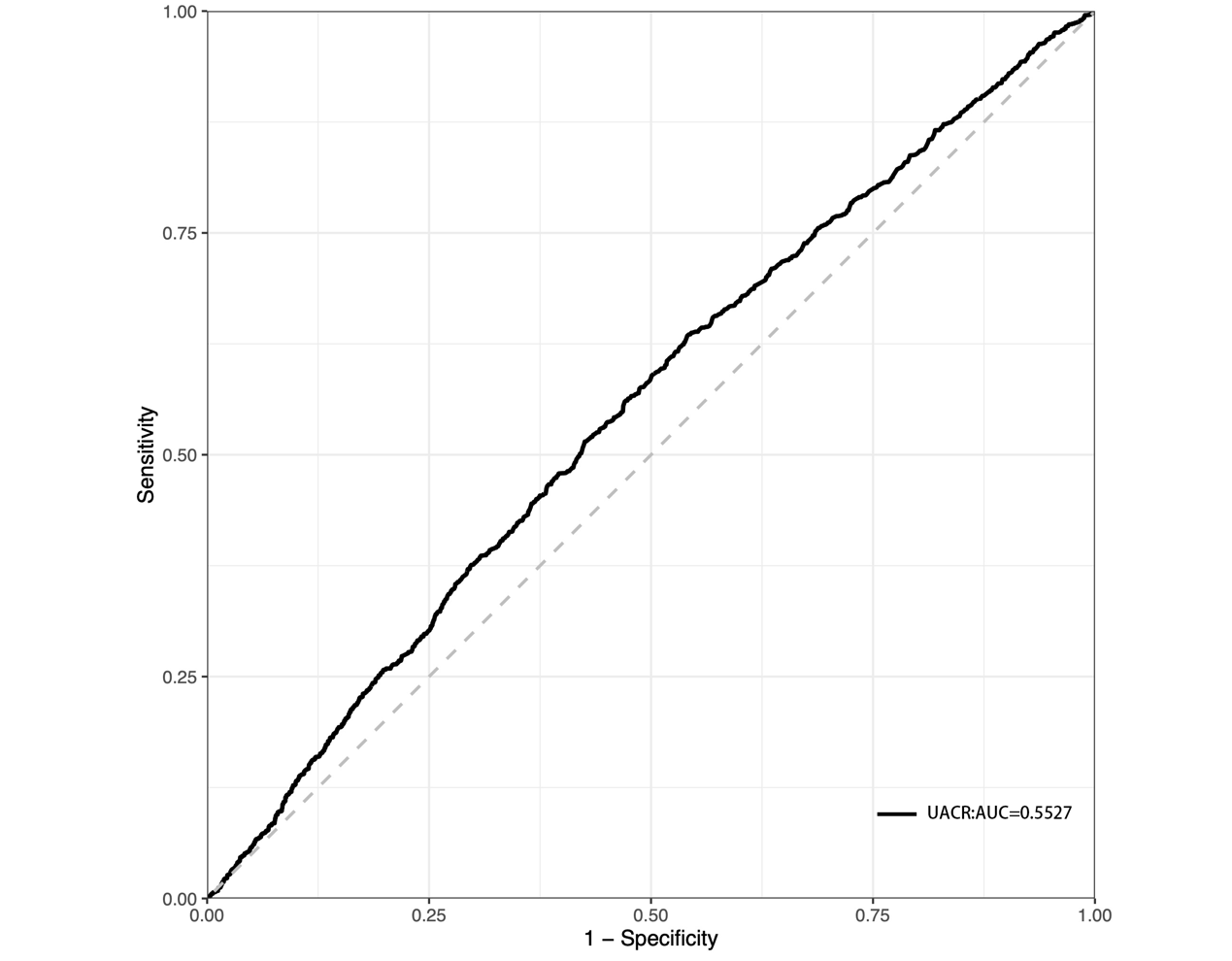
**
